# Predicting metachronous liver metastasis in patients with colorectal cancer: development and assessment of a new nomogram

**DOI:** 10.1186/s12957-022-02558-6

**Published:** 2022-03-12

**Authors:** Mengdi Hao, Huimin Li, Kun Wang, Yin Liu, Xiaoqing Liang, Lei Ding

**Affiliations:** 1 Department of Oncology Surgery, Beijing Shijitan Hospital, Capital Medical University, Beijing, China; 2 Department of Oncology Surgery, Ninth School of Clinical Medicine, Peking University, Beijing, China

**Keywords:** Colorectal cancer, Metachronous liver metastasis, Nomogram, Risk factor

## Abstract

**Background:**

We aimed to develop and validate a nomogram model, which could predict metachronous liver metastasis in colorectal cancer within two years after diagnosis.

**Methods:**

A retrospective study was performed on colorectal cancer patients who were admitted to Beijing Shijitan Hospital from January 1, 2016 to June 30, 2019. The least absolute shrinkage and selection operator (LASSO) regression model was used to optimize feature selection for susceptibility to metachronous liver metastasis in colorectal cancer. Multivariable logistic regression analysis was applied to establish a predictive model through incorporating features selected in the LASSO regression model. C-index, receiver operating characteristic (ROC) curve, calibration plot, and decision curve analysis (DCA) were employed to assess discrimination, distinctiveness, consistency with actual occurrence risk, and clinical utility of candidate predictive model. Internal validation was assessed with bootstrapping method.

**Results:**

Predictors contained in candidate prediction nomogram included age, CEA, vascular invasion, T stage, N stage, family history of cancer, and KRAS mutation. This model displayed good discrimination with a C-index of 0.787 (95% confidence interval: 0.728–0.846) and good calibration, whereas area under the ROC curve (AUC) of 0.786. Internal validation obtained C-index of 0.786, and AUC of validation cohort is 0.784. Based on DCA, with threshold probability range from 1 to 60%; this predictive model might identify colorectal cancer metachronous liver metastasis to achieve a net clinical benefit.

**Conclusion:**

We have developed and validated a prognostic nomogram with good discriminative and high accuracy to predict metachronous liver metastasis in CRC patients.

## Background

Liver metastasis, as the most commonly involved organ by colorectal cancer, has been recognized as the leading causes of death. The WHO announced more than 1.9 million new cases worldwide in 2020 [1], of which nearly half of patients develop liver metastasis during the course of the disease. Liver metastasis, with high incidence and mortality, has become the primary determinant of poor prognosis and frequent recurrence of colorectal cancer [2–4]. Although primary tumor and liver metastasis can be detected by preoperative thoraco-abdominal computed tomography (CT) in time, and these patients can be treated with surgical intervention, neoadjuvant chemoradiotherapy, or adjuvant chemoradiotherapy, a significant proportion of colorectal cancer cases, approximately 15–25%, would inevitably develop liver metastasis during follow-up after primary tumor resection [5–7]. Metachronous liver metastasis (MLM) is defined when liver involvement occurs after diagnosis/operation of primary colorectal cancer (cut-off point). Both prognosis and quality of life of patients with colorectal cancer who have undergone MLM are inferior to those with colorectal localized tumors, regardless of secondary resection, or adjuvant chemotherapy or targeted therapy. However, molecular mechanism of MLM is not yet clear, its pathogenesis can be affected by clinicopathological features, such as histological patterns, preoperative tumor markers, as well as genetics/epigenetics.

Nomograms are mainly used for risk prediction and prognostic evaluation. Currently, nomograms are widely applied in clinical studies on cancer patients. By assigning scores to various predictive factors, calculating and evaluating the probability of dependent variables, complex regression analysis can be converted into visual graphics [8–11]. A large number of studies have focused on potential factors that cause colorectal cancer and contribute to distant metastasis, in particular liver metastasis. For patients who have undergone primary resection without liver metastasis as demonstrated by preoperative imaging, postoperative MLM significantly affects prognosis and quality of life.

Therefore, this study aims to establish a nomogram model to evaluate patients with colorectal cancer with a high-risk score for liver metastasis, so as to help clinicians predict prognosis and provide a more personalized follow-up plan for colorectal cancer patients after surgery.

## Methods

### Study populations

**Fig. 1:**
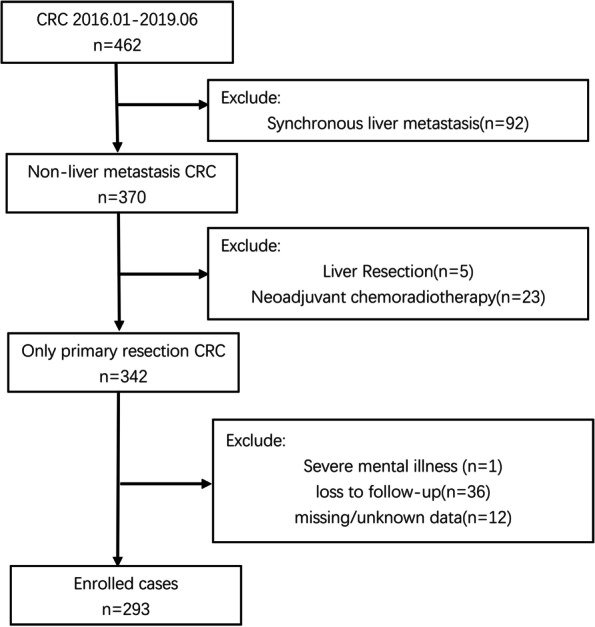
Study flowchart

This investigation was approved by Ethics Committee of Beijing Shijitan Hospital (#[ sjtky11-1x-2021(106)]) and performed in accordance with the Declaration of Helsinki. Clinical data of patients diagnosed in the Department of Oncology at Beijing Shijitan Hospital from January 1, 2016 to June 30, 2019 were retrospectively collected, including demographics (gender, age, ethnicity), family history of cancer, body-mass index (BMI), preoperative serum indicators [albumin (ALB), alpha fetoprotein (AFP), carcinoembryonic antigen (CEA), carbohydrate antigen 19-9 (CA19-9)], primary tumor location, pathological features (differentiation grade, the maximum diameter, tissue infiltration, vascular invasion, perineural invasion, T, N, Dukes’ staging), as well as gene expression profiling (e.g., KRAS, NRAS). A total of 293 patients were enrolled after screening (Fig. 1). All enrolled patients were followed up for 2 years. Follow-up was terminated for patients with recurrent liver metastasis identified by thoraco-abdominal CT after primary tumor resection, and the remaining patients were followed up until June 30, 2021. Results of the last thoraco-abdominal CT were recorded. The occurrence of MLM within 2 years after diagnosis/operation was defined as an unfavorable event. Written informed consent was obtained from each subject.

### Inclusion and exclusion criteria

The inclusion criteria specified patients (1) evaluated as undergoing curative resection without any preoperative adjuvant therapy, (2) pathologically diagnosed as adenocarcinoma, (3) no signs of liver metastasis on preoperative imaging, and (4) no history of other malignancies in the past 5 years. This study excluded patients who (1) were diagnosed as synchronous liver metastasis before resection, (2) underwent both bowel and metastasis resection, (3) received adjuvant therapy such as neoadjuvant chemotherapy or radiotherapy before surgery, (4) had serious cardiac and/or brain diseases, (5) had a history of mental illness, or a family history of mental illness, (6) were not regularly reviewed with thoraco-abdominal CT after operation, (7) had unclear status of liver metastasis, (8) were lost to follow-up, and (9) had missed clinical data.

### Statistical analysis

All data were sorted and expressed as *n* (%). Categorical variables were analyzed using a chi-square test (or Fisher’s exact test under specific conditions) when comparing differences between groups. Statistical analysis was performed with the SPSS 26.0 statistical package (SPSS Inc., Chicago, IL, USA) and R software (Version:4.0.3 http://www.R-project.org). The LASSO [12–14] was used to select candidate risk factors for colorectal cancer MLM. Non-zero coefficient features were selected from LASSO regression models to describe odds ratios (ORs), 95% confidence intervals (CIs), and *p* values of selected predictors. Multivariate logistic regression analysis was used to verify selected predictors, combined with results of LASSO regression analysis to establish a prediction model and to draw a nomogram model to predict individual risk of MLM in colorectal cancer. All statistical tests were two-sided, and a *p* < 0.05 was considered statistically significant. Harrell’s C-index was calculated to quantify discrimination performance, the larger the C index, the stronger the predictive ability of the model [15, 16]. Receiver operating characteristic (ROC) curve was plotted. The area under the curve (AUC) was calculated to evaluate predictive value for MLM [15]. We applied 1000 bootstrap resamples to establish a calibration curve, so that consistency between predicted value and actual value could be assessed. In order to explore clinical application value of candidate model, DCA was used to calculate net benefit under the probability of each risk threshold [17]. Finally, candidate model was verified internally, with its credibility determined by C-index, AUC and calibration curve.

## Results

### Clinicopathologic characteristics

A total of 293 patients with colorectal cancer were analyzed, including 171 males and 122 females. The elderly (> 60 years old) cases accounted for a large proportion (232/293, 79.18%). Pre-operative serum CEA level was normal in 132 cases, borderline in 21 cases, and increased in 140 cases, respectively. The maximum diameter of primary tumor was ≤ 5 cm in 173 cases, whereas > 5 cm in 120 cases, respectively. Postoperative pathological stages were categorized as pT_1_ in 22 cases, pT_2_ in 65 cases, pT_3_ in 130 cases, whereas pT_4_ in 76 cases. Lymph node was involved (pN+) in 210 patients, whereas spared (pN0) in 83 patients. According to presence vs. absence of liver metastasis on abdominal CT within 2 years, all patients were divided into two groups: MLM (*n* = 75) and non-MLM (*n* = 218). According to baseline characteristics of the included patients, we observed that there were significant differences in CEA, vascular invasion, pT stage, Dukes’ staging, KRAS mutation (*p* < 0.05), and no significant differences in other factors. The basic demographic and clinicopathologic characteristics were presented in Table 1.

**Table 1:** Differences between demographic and clinicopathologic characteristics of MLM and non-MLM groups

Clinicopathologic Characteristics	*n* (%)			*P*
	MLM group (***n*** = 75)	Non-MLM group (***n*** = 218)	Total (***n*** = 293)	
Gender				.306
Male	40 (53.33)	131 (60.09)	171 (58.36)	
Female	35 (46.67)	87 (39.91)	122 (41.64)	
Age (years)				.595
≤60	14 (18.67)	47 (21.56)	61 (20.82)	
>60	61 (81.33)	171 (78.44)	232 (79.18)	
Family history of cancer				.246
No	21 (28.00)	77 (35.32)	98 (33.45)	
Yes	54 (72.00)	141 (64.68)	195 (66.56)	
BMI				.965
≤ 25	47 (62.67)	136 (62.39)	183 (62.46)	
>25	28 (37.33)	82 (37.61)	110 (37.54)	
ALB				.554
Normal	23 (30.67)	75 (34.40)	98 (33.45)	
Abnormal	52 (69.33)	143 (65.60)	195 (66.55)	
CEA				.001
Normal	21 (28)	111 (50.92)	132 (45.05)	
Abnormal	44 (58.67)	96 (44.04)	140 (47.78)	
Borderline	10 (13.33)	11 (5.05)	21 (7.17)	
AFP				.973
Normal	74 (98.67)	213 (97.71)	287 (97.95)	
Abnormal	1 (1.33)	5 (2.29)	6 (2.05)	
CA199				.683
Normal	5 (6.67)	12 (5.50)	17 (5.80)	
Abnormal	65 (86.67)	196 (89.91)	261 (89.08)	
Borderline	5 (6.67)	10 (4.59)	15 (5.12)	
Tumor primary location				.510
Ascending colon	22 (29.33)	63 (28.90)	85 (29.01)	
Transverse colon	4 (5.33)	8 (3.67)	12 (4.10)	
Descending colon	30 (40.00)	70 (32.11)	100 (34.13)	
Sigmoideum	13 (17.33)	57 (26.14)	70 (23.89)	
Boundary	6 (8.00)	20 (9.17)	26 (8.87)	
Differentiation degree				.113
High	6 (8.00)	15 (6.88)	21 (7.17)	
High–medium	3 (4.00)	32 (14.68)	35 (11.95)	
Medium	51 (68.00)	143 (65.60)	194 (66.21)	
Medium–low	11 (14.67)	20 (9.17)	31 (10.58)	
Low	4 (5.33)	8 (3.67)	12 (4.10)	
Max (cm)				.727
≤ 5	43 (57.33)	130 (59.63)	173 (59.04)	
>5	32 (42.67)	88 (40.37)	120 (40.96)	
Tissue infiltration				.312
No	24 (32)	84 (38.53)	108 (36.86)	
Yes	51 (68)	134 (61.67)	185 (63.14)	
Vascular invasion				<.001
No	40 (53.33)	165 (75.69)	205 (69.97)	
Yes	35 (46.67)	53 (24.32)	88 (30.03)	
Perineural invasion				.937
No	53 (70.67)	153 (70.19)	206 (70.31)	
Yes	22 (29.33)	65 (29.82)	87 (29.69)	
pT				.002
pT_1_	1 (1.33)	21 (9.63)	22 (7.51)	
pT_2_	12 (16.00)	53 (24.31)	65 (22.18)	
pT_3_	32 (42.67)	98 (44.95)	130 (44.37)	
pT_4_	30 (40.00)	46 (21.10)	76 (25.94)	
pN				.064
pN_0_	15 (20.00)	68 (31.19)	83 (28.33)	
pN_+_	60 (80.00)	150 (68.81)	210 (71.67)	
Dukes				.008
A	13 (17.33)	74 (33.94)	87 (29.69)	
B	14 (18.67)	52 (23.85)	66 (22.52)	
C	46 (61.33)	86 (39.45)	132 (45.05)	
D	2 (2.67)	6 (2.75)	8 (2.73)	
Kras mutation				<.001
Wild	45 (60.00)	176 (80.73)	221 (75.43)	
Mutation	30 (40.00)	42 (19.27)	72 (24.57)	
Nras mutation				.802
Wild	48 (64.00)	143 (65.60)	191 (65.19)	
Mutation	27 (36.00)	75 (34.40)	102 (34.81)	

*MLM group* metachronous liver metastasis group, *non*-*MLM group* non-metachronous liver metastasis group

### Feature selection

**Fig. 2:**
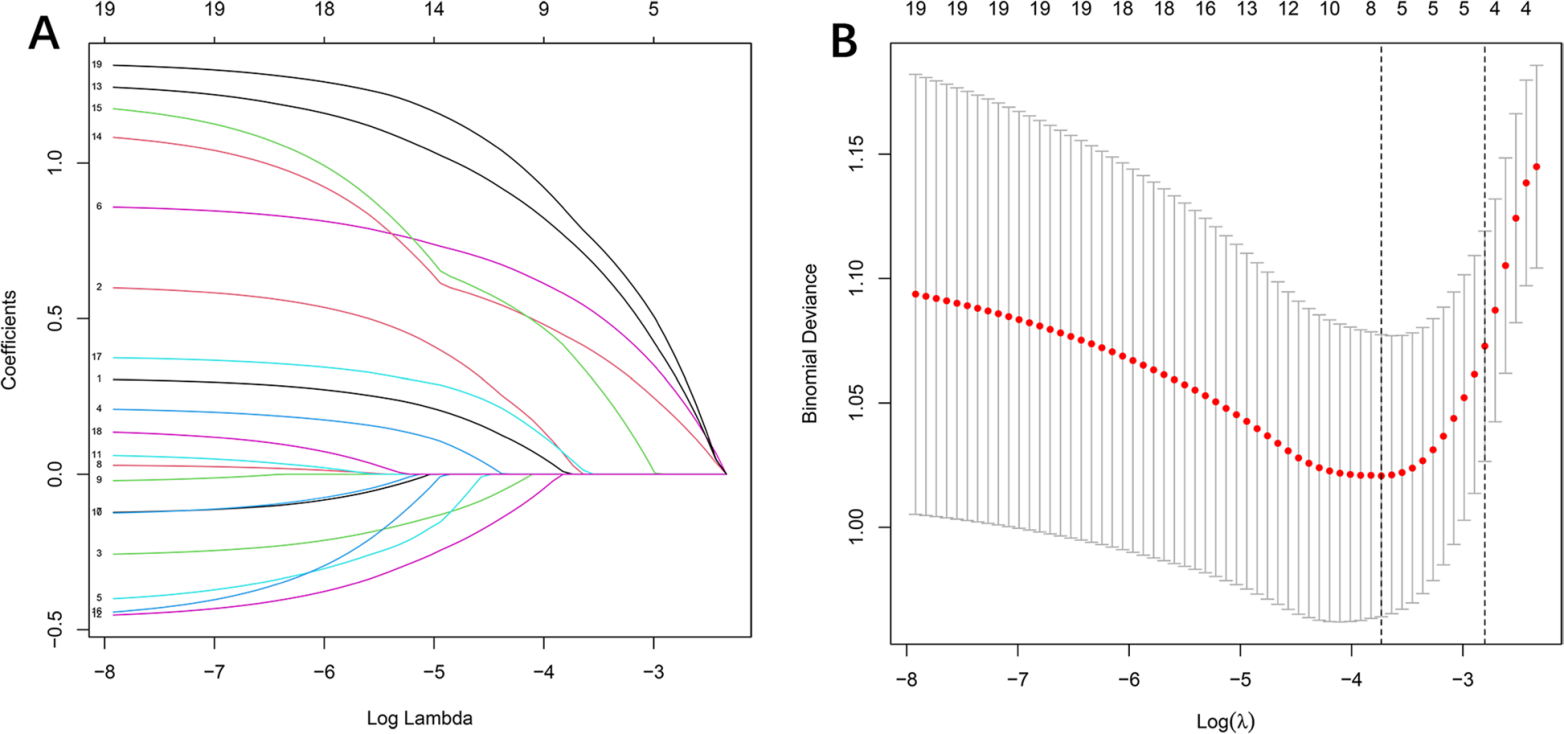
Demographic and clinicopathologic feature selection using the LASSO binary logistic regression model. **A** Screen the included 19 clinical variables and a coefficient profile plot was produced against the log(lambda) sequence. **B** The smallest lambda is obtained by tenfold cross-validation. When the smallest lambda is equal to 0.024, lasso regression retains 7 non-zero coefficient independent variables

Among 19 variables, 7 potential risk factors with nonzero coefficients were identified by LASSO regression analysis, including age, CEA, vascular invasion, T stage, N stage, family history of cancer, and KRAS mutation (Fig. 2A, B).

Multivariate logistic regression analysis identified CEA level, vascular invasion, pT_4_, pN_+_, KRAS mutation as independent risk factors for MLM (Table 2).

**Table 2:** Prediction factors for metachronous liver metastasis in CRC

Intercept and variable	Prediction model		
	Odds ratio (OR)	95% CI	***P***-value
Age			
≤ 60	1		
>60	1.787	0.831–4.059	0.149
CEA			
Normal	1		
Abnormal	2.071	1.096–3.995	0.027
Borderline	5.440	1.890–15.800	0.002
Vascular invasion			
No	1		
Yes	3.160	1.702–5.951	<0.001
pT			
pT_1_	1		
pT_2_	3.018	0.504–58.175	0.314
pT_3_	4.724	0.858–88.602	0.146
pT_4_	10.104	1.816–190.352	0.031
pN			
pN_0_	1		
pN+	2.353	1.177–4.953	0.019
Family history of cancer			
No	1		
Yes	1.492	0.788–2.905	0.228
Kras mutation			
Wild	1		
Mutation	3.658	1.864–7.315	<0.001

### Development of an individualized prediction model

**Fig. 3:**
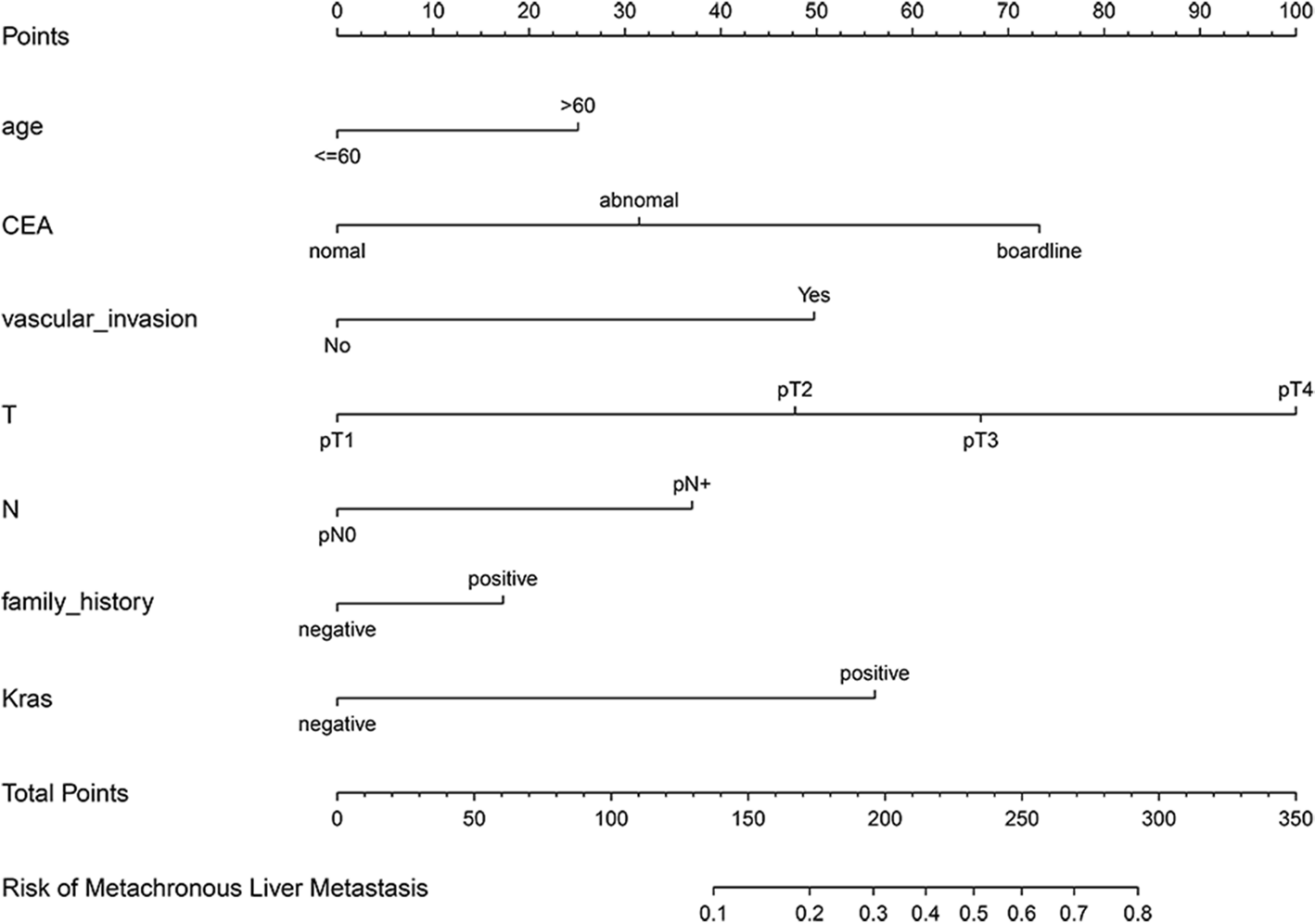
Nomogram for predicting metachronous liver metastasis in patients with colorectal cancer. The nomogram was developed in the cohort, with age, pre-op CEA level, vascular invasion, T stage, N stage, family history of tumor, Kras gene

Based on regression analysis, a nomogram model predicting risk factors for MLM of colorectal cancer was developed (Fig. 3), with C-index of 0.787 (95% CI: 0.728–0.846).

### Performance and validation of nomograms for MLM prediction

**Fig. 4:**
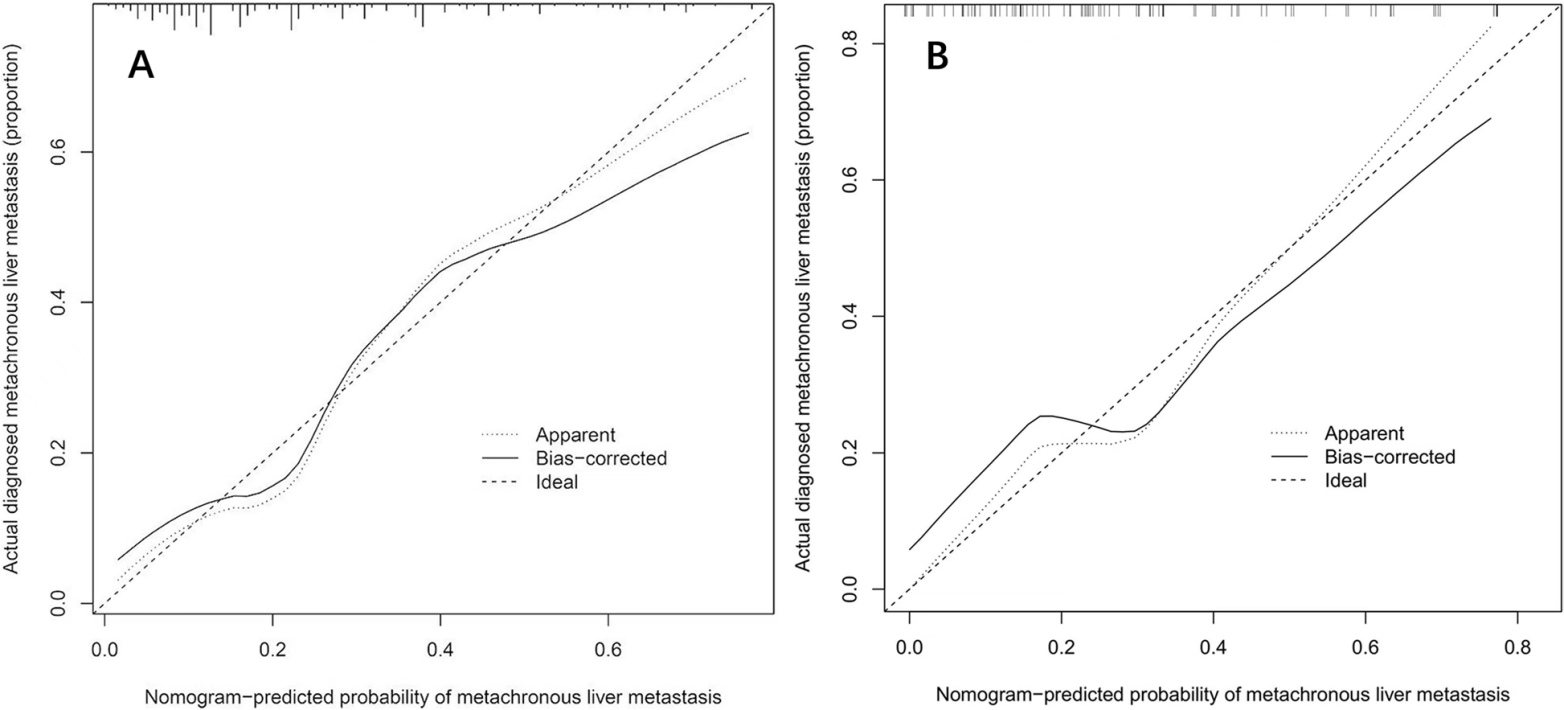
Calibration curves of the MLM nomogram prediction in the cohort. **A** Calibration curves of training cohort. **B** Calibration curves of validation cohort. Notes: The *x*-axis represents the predicted MLM risk. The *y*-axis represents the actual diagnosed MLM

**Fig. 5:**
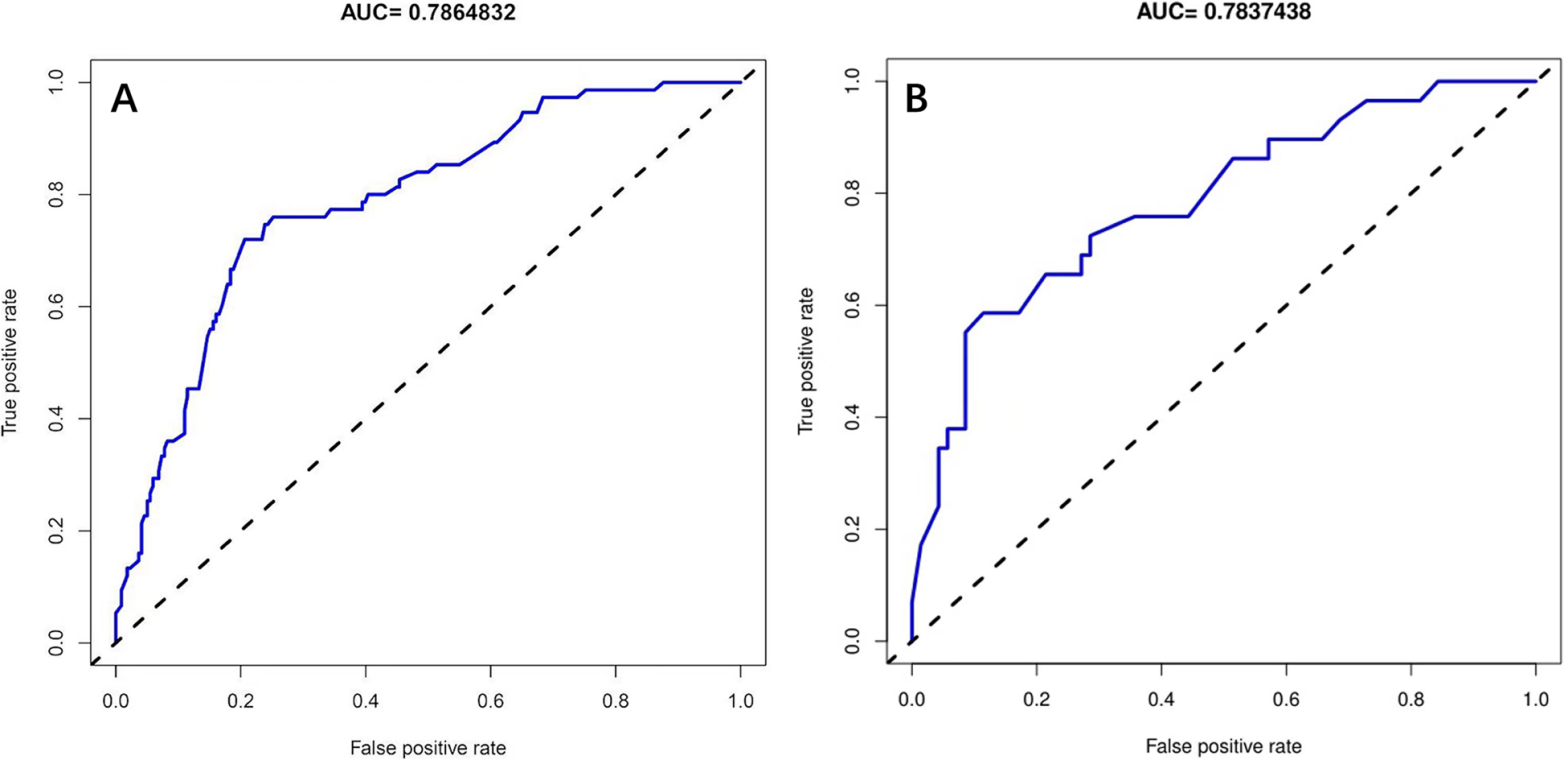
Receiver operating characteristic (ROC) curve analysis for MLM. Comparisons of the predictive values of the nomogram models and clinicopathological risk factors for metachronous liver metastasis according to ROC analysis. AUC = 0.786 in training cohort (**A**) and AUC = 0.784 in validation cohort (**B**), both AUC > 0.7

**Fig. 6:**
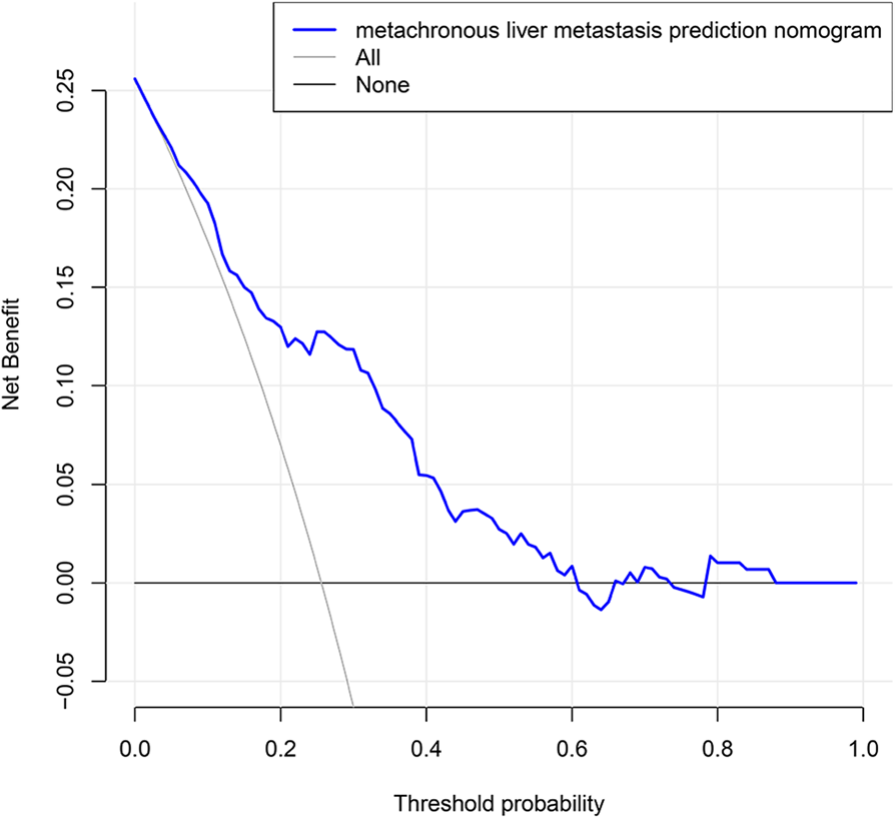
Decision curve analysis for the MLM nomogram. The *Y*-axis is net income. The dotted line represents the MLM nomogram. when the threshold probability is > 1% and < 60%, using this predictive model to identify colorectal cancer metachronous liver metastasis could achieve a net clinical benefit

In this study, calibration curve used to predict MLM in patients with colorectal cancer exhibited good consistency (Fig. 4A). In order to evaluate discriminative and predictive capability of candidate nomogram model, ROC curve was drawn, with AUC of 0.786 (Fig. 5A). Based on DCA (Fig. 6), with the threshold probability ranged from 1 to 60%, using this predictive model to identify colorectal cancer MLM could achieve a net clinical benefit. In addition, bootstrap testing was applied to validate this model. C-index was 0.786 (95% CI 0.68702–0.88498), further calibration curve (Fig. 4B) and ROC curve (AUC 0.784) were drawn (Fig. 5B), which proved good reliability of candidate predictive model.

## Discussion

Colorectal cancer, as the third most common malignancy worldwide, has become a hot topic for clinical and scientific research in recent years [1]. Especially liver metastasis, the most common cause of death in colorectal cancer, poses a great threat to public health. For patients with no signal of liver metastasis at initial diagnosis, if liver metastasis occurs after primary resection, decreased survival rate and quality of life will be expected. Therefore, it is particularly important to accurately evaluate each patient’s condition in all aspects before surgery and timely detect MLM, in order to adjust treatment strategy at an early stage and improve prognosis.

How do we define MLM? Currently, there is no international consensus on the time point for delineating ‘synchronous’ and ‘metachronous’ metastasis. Most of the studies did not clearly distinguish between ‘synchronous’ and ‘metachronous’ liver metastasis. And in the few studies that did distinguish between ‘synchronous’ and ‘metachronous,’ the authors included the time of diagnosis of liver metastasis and the interval of 3, 6, and 12 months after the initial colorectal cancer diagnosis/surgery as reference points in the MLM group, respectively [18–23], which were allowed. A significant number of studies are more supportive of the initial colorectal cancer diagnosis/surgery as the dividing line between synchronous and MLM than the former [23–25]. Among them, Engstrand et al. [25] summarized previous studies and assessed prognosis at different defined time points, supporting the initial diagnosis/surgery as the standard cut-off point between the ‘synchronous’ and ‘metachronous’ groups. The present study adopts this view and uses it as a reference for the grouping of the synchronous and MLM groups.

At present, nomogram model has been widely used in clinical medicine, particularly in colorectal surgery [26, 27]. In view of the high morbidity and mortality characteristics of colorectal cancer, various predictive models focusing on the prognosis of colorectal cancer have been developed in recent years [28–30], including a prognostic model related to liver metastasis [31–33]. Time-dependent factors can effectively predict patient survival. In addition, there has been an increasing interest in exploring the risk of developing liver metastasis. Ding et al. [34] applied the nomogram model to show us the risk factors for liver metastasis from colorectal neuroendocrine neoplasm. Mo et al. [35] analyzed the specific distant metastatic sites of stage I–IV colorectal cancer by univariate and multivariate logistic regression analysis, supporting the application of the nomogram model based on clinicopathological features to predict the metastatic sites of colorectal cancer, while confirming to us that sex, tumor site, grade, age, histological type, tumor size, T stage, N stage, and lymph node harvested were important risk factors for liver metastasis from colorectal cancer. Tang et al. [36] analyzed clinical data from the SEER database of 203,998 colorectal cancer patients to establish a nomogram to predict synchronous liver metastasis from colorectal cancer and concluded that male, black, uninsured status, left colon, T4/T1, bone, and lung metastasis were positively associated with the risk of synchronous liver metastasis.

To our knowledge, more reports have focused on the prognosis of liver metastasis from colorectal cancer and the impact of surgical treatment on the survival of patients with synchronous liver metastasis from colorectal cancer, and few studies have focused on MLM from colorectal cancer and no corresponding nomogram models have been developed. Therefore, this study focused on the risk and prognostic factors of MLM and developed and validated a nomogram model to predict the likelihood and risk factors of MLM in colorectal cancer during the high-risk time period for the development of MLM, i.e., 2 years after surgery.

By screening variables and assigning scores to those variables, nomogram visualizes data from multivariable regression analysis and individually predicts susceptibility to clinical events. In this study, LASSO regression analysis was adopted for variable selection. The LASSO regression model can not only combine selected features into radiomic features, but also check correlation between predicted factors, reduce selection bias, and optimize prediction [12, 37, 38]. Of 21 clinical factors, 7 variables were selected by LASSO regression analysis. Based on multiple logistic regression model, CEA level, vascular invasion, pT4, pN+, and KRAS mutation were independent risk factors for MLM of colorectal cancer. Combining the above two models, we established and verified a nomogram model for predicting potential risk of MLM within two years after diagnosis/operation.

As is known to all, compared with the young, the elderly is more likely to be diagnosed as malignant tumor, colorectal cancer is no exception. Studies have considered the mean age of the MLM group was younger than that of the synchronous liver metastasis group [39]. The latter is outside this study, we therefore compared the MLM cohort and non-MLM cohort of age, 18.67% of the patients in the MLM cohort are younger than 60 years old, and 21.56% were non-elderly patients in the non-MLM cohort. Age did not differ, but met the criteria for inclusion in the nomogram model, and we included it in the prediction model, with a score of 25 for the risk factor of > 60 years (Fig. 3), which is significant for predicting MLM.

CEA is mainly cleared in the liver [40], and abnormal liver function caused by tumor implantation may lead to the increase of serum CEA. Similar to previous studies, CEA is recognized as an important tumor marker for colorectal cancer. Pre-operative CEA and post-operative CEA suggest an association with systemic disease [41], increased pre-operative CEA accelerating metastasis, and spread of tumors after surgery [42]. Generally, increase in serum CEA level may be associated with liver metastasis of colorectal cancer [43–45]. Chuang et al. [46] retrospectively analyzed 1099 patients who underwent curative resection MLM of colorectal cancer by conducting univariate and multivariate analyses. Interestingly, preoperative serum CEA level, positive tumor depth, lymph node metastasis, and vascular invasion predicted MLM after curative resection. In addition, Mohr et al. [47] observed consistent trends. Although previous studies have suggested that postoperative serum CEA is a risk factor for liver metastasis of colorectal cancer [48], controversy remains inconclusive. In our study, patients with high or borderline levels of preoperative serum CEA are more likely to develop MLM within 2 years than those with normal levels, which is not contrary to actual clinical experience.

Genotypic differences of the primary tumor lead to differences in tumor behavior, causing MLM or synchronous liver metastasis [49]. Among the many colorectal cancer genes, RAS genetic alteration is the only recognized prognostic indicator of colorectal cancer. The KRAS mutation rate can reach 25–52% [50–52]. Previous studies [53] have shown that KRAS codon 13 mutation is an independent factor for metachronous distant metastasis of colorectal cancer, but there is no conclusive evidence for MLM currently, so this study focuses on the effect of RAS genes on MLM. Of the 293 patients enrolled in our cohort, 72 carried KRAS mutations (24.57%). 40% of patients with MLM within 2 years harbored KRAS mutations, which is consistent with other centres. Nras mutation occurred in 34.81%, and of the 75 patients with metachronous liver metastasis, 64% were Nras wild type, and 36% were Nras mutation, with a lower probability of Nras mutation in patients with metachronous liver metastasis compared to Kras. The absence of statistically significant Nras mutation in our study cannot be ruled out as a limitation of the limited sample size. LASSO regression screened out KRAS gene as a predictor of MLM. Multivariate logistic regression verified KRAS mutation as an independent risk factor for liver metastasis of colorectal cancer (*p* value < 0.001) and was included in the nomogram prediction model, with Kras positivity scoring 58 points in the model, effectively predicting metachronous liver metastasis.

Currently, tumor-node-metastasis (TNM) is a well-accepted staging system for colorectal cancer, with invasion depth and lymph node involvement closely related to liver metastasis [54–56]. Khan et al. [48] retrospectively analyzed the clinicopathological data of 434 patients with rectal cancer, and concluded that T staging and lymph node metastasis were related to the MLM of rectal cancer. This is consistent with the opinion of Chuang et al. [46]. A recent Italian study highlighted that lymph node ratios (ratio of positive lymph nodes to the total number of lymph nodes retrieved) can be a predictor of MLM after surgery when lymph nodes are sampled in sufficient numbers [57]. In addition, lymph nodes are considered to be independent risk factors for vascular invasion [58], and the combined action of the three factors can accelerate the progression of postoperative MLM. There are, of course, still a few opposing views that support the different subtypes of lymph node metastasis and distant metastasis, and lymph node status should not be treated as a precursor of distant metastasis [39, 56]. Due to the limitations of the study subjects and the diverse molecular subtypes of colorectal cancer patients, it is difficult to independently confirm whether T stage, N stage, and vascular invasion promote or inhibit liver metastasis. By Lasso regression analysis and logistic regression analysis, pT, pN, and vascular invasion were considered as the more important predictors in candidate nomogram model, with pT4, pN+, and positive vascular invasion being independent risk factors for MLM from colorectal cancer (*p* < 0.05), a view that would be supported by the majority of studies.

Imaging evaluation of liver metastasis is the mainstay to assess progression of colorectal cancer in clinical practice, especially CT and MRI, which are the most commonly used auxiliary methods for colorectal cancer patients. Thoraco-abdominal CT is mainly used to evaluate the depth of local invasion and distant staging. Although MRI can make up for the limited accuracy of CT scan and further stage distant metastasis, due to the limitations of objective factors such as cost and time cost, no matter preoperative diagnosis or postoperative review, thoraco-abdominal CT is still the most commonly used imaging examination for the diagnosis of distant metastasis of colorectal cancer [59, 60]. Therefore, liver metastasis with thoraco-abdominal CT was regarded as an outcome event in this study. If tumors have reached pT4 and involved lymph nodes when undergoing curative resection, small liver metastatic lesions cannot be ruled out. Thus, accurate assessment can help identify potential risk of MLM in patients with colorectal cancer, and specify individual follow-up plan. Simultaneously, high-risk patients can receive more effective treatment. This prediction model can be used as an auxiliary method for imaging to jointly predict MLM of colorectal cancer.

There are some limitations in the present study. First, only patients admitted to Beijing Shijitan hospital were recruited. Second, it is difficult to include all risk factors affecting liver metastasis, so our results may be biased to some extent. In addition, patients with colorectal cancer generally receive chemotherapy after surgery. Due to individual differences in sensitivity to chemotherapy, development of liver metastasis may be affected by different drugs. However, there is currently no definite evidence that chemotherapy has an impact on our observation. Third, due to the limitation of follow-up time, we only predicted the risk factors for MLM within two years, although this is the most common time for the occurrence of MLM, it is still necessary to further study the risk factors for MLM at different times in the additional study, which will provide greater help for doctors to predict liver metastasis from colorectal cancer. Finally, although bootstrap test was used for internal validation of candidate model, external validation was not performed. Therefore, its applicability to colorectal cancer in other regions and countries remains unknown, and more extensive external verification should be carried out.

## Conclusion

We have established a nomogram model for predicting potential risk of MLM from colorectal cancer, which has good discrimination and high accuracy. This model may help assess susceptibility to MLM in patients with colorectal cancer after surgery and develop individualized treatment and follow-up plans. This model predicts clinically liver metastasis, and thus provides an important reference for screening.

## Data Availability

The datasets generated and/or analyzed during the current study are not publicly available but are available from the corresponding author on reasonable request.

## Abbreviations

MLM: Metachronous liver metastasis
LASSO: Least absolute shrinkage and selection operator
ROC: Receiver operating characteristic
DCA: Decision curve analysis
AUC: The area under the curve
CT: Computed tomography
BMI: Body-mass index
ALB: Albumin
AFP: Alpha fetoprotein
CEA: Carcinoembryonic antigen
CA19-9: Carbohydrate antigen 19-9
ORs: Odds ratios
CIs: Confidence intervals
TNM: Tumor-node-metastasis

## References

[1] Sung H, Ferlay J, Siegel RL, Laversanne M, Soerjomataram I, Jemal A, Bray F. Global cancer statistics 2020: GLOBOCAN estimates of incidence and mortality worldwide for 36 cancers in 185 countries. CA Cancer J Clin. 2021;71(3):209-249. 10.3322/caac.21660 33538338

[2] Stewart CL, Warner S, Ito K, Raoof M, Wu GX, Kessler J (2018). Cytoreduction for colorectal metastases: liver, lung, peritoneum, lymph nodes, bone, brain. When does it palliate, prolong survival, and potentially cure?. Curr Probl Surg.

[3] Wu L, Fu J, Chen Y, Wang L, Zheng S (2020). Early T stage is associated with poor prognosis in patients with metastatic liver colorectal cancer. Front Oncol.

[4] Siegel RL, Miller KD, Goding Sauer A, Fedewa SA, Butterly LF, Anderson JC (2020). Colorectal cancer statistics, 2020. CA Cancer J Clin.

[5] Giannis D, Sideris G, Kakos CD, Katsaros I, Ziogas IA (2020). The role of liver transplantation for colorectal liver metastases: a systematic review and pooled analysis. Transplant Rev (Orlando).

[6] Gregoire E, Hoti E, Gorden DL, de la Serna S, Pascal G, Azoulay D (2010). Utility or futility of prognostic scoring systems for colorectal liver metastases in an era of advanced multimodal therapy. Eur J Surg Oncol.

[7] Colloca GA, Venturino A, Guarneri D (2020). Different variables predict the outcome of patients with synchronous versus metachronous metastases of colorectal cancer. Clin Transl Oncol.

[8] Tsilimigras DI, Xiang JX, Zhang XF, Pawlik TM (2020). ASO author reflections: a nomogram to predict recurrence after curative-intent resection for neuroendocrine liver metastasis. Ann Surg Oncol.

[9] Kluth LA, Black PC, Bochner BH, Catto J, Lerner SP, Stenzl A (2015). Prognostic and prediction tools in bladder cancer: a comprehensive review of the literature. Eur Urol.

[10] Ó Hartaigh B, Gransar H, Callister T, Shaw LJ, Schulman-Marcus J, Stuijfzand WJ, Valenti V, Cho I, Szymonifka J, Lin FY (2018). Development and validation of a simple-to-use nomogram for predicting 5-, 10-, and 15-year survival in asymptomatic adults undergoing coronary artery calcium scoring. JACC Cardiovasc Imaging.

[11] Lo SN, Ma J, Scolyer RA, Haydu LE, Stretch JR, Saw RPM (2020). Improved risk prediction calculator for sentinel node positivity in patients with melanoma: the melanoma institute australia nomogram. J Clin Oncol.

[12] Sauerbrei W, Royston P, Binder H (2007). Selection of important variables and determination of functional form for continuous predictors in multivariable model building. Stat Med.

[13] Huang YQ, Liang CH, He L, Tian J, Liang CS, Chen X (2016). Development and validation of a radiomics nomogram for preoperative prediction of lymph node metastasis in colorectal cancer. J Clin Oncol.

[14] Friedman J, Hastie T, Tibshirani R (2010). Regularization paths for generalized linear models via coordinate descent. J Stat Softw.

[15] Liu W, Zhang W, Xu Y, Li YH, Xing BC. A prognostic scoring system to predict survival outcome of resectable colorectal liver metastases in this modern era. Ann Surg Oncol. 2021;28(12):7709-7718. 10.1245/s10434-021-10143-6 34023948

[16] Pencina MJ, D'Agostino RB (2004). Overall C as a measure of discrimination in survival analysis: model specific population value and confidence interval estimation. Stat Med.

[17] Vickers AJ, Elkin EB (2006). Decision curve analysis: a novel method for evaluating prediction models. Med Decis Mak.

[18] Bredt LC, Rachid AF (2014). Predictors of recurrence after a first hepatectomy for colorectal cancer liver metastases: a retrospective analysis. World J Surg Oncol.

[19] Hackl C, Neumann P, Gerken M, Loss M, Klinkhammer-Schalke M, Schlitt HJ (2014). Treatment of colorectal liver metastases in Germany: a ten-year population-based analysis of 5772 cases of primary colorectal adenocarcinoma. BMC Cancer.

[20] Angelsen JH, Horn A, Sorbye H, Eide GE, Loes IM, Viste A (2017). Population-based study on resection rates and survival in patients with colorectal liver metastasis in Norway. Br J Surg.

[21] John SK, Robinson SM, Rehman S, Harrison B, Vallance A, French JJ (2013). Prognostic factors and survival after resection of colorectal liver metastasis in the era of preoperative chemotherapy: an 11-year single-centre study. Dig Surg.

[22] Nakayama I, Suenaga M, Wakatsuki T, Ichimura T, Ozaka M, Takahari D (2015). Safety, tolerability, and efficacy of oxaliplatin-based adjuvant chemotherapy after curative resection of hepatic or extrahepatic metastases of Stage IV colorectal cancer. Cancer Chemother Pharmacol.

[23] Ng WW, Cheung YS, Wong J, Lee KF, Lai PB (2009). A preliminary analysis of combined liver resection with new chemotherapy for synchronous and metachronous colorectal liver metastasis. Asian J Surg.

[24] Dexiang Z, Li R, Ye W, Haifu W, Yunshi Z, Qinghai Y (2012). Outcome of patients with colorectal liver metastasis: analysis of 1,613 consecutive cases. Ann Surg Oncol.

[25] Engstrand J, Stromberg C, Nilsson H, Freedman J, Jonas E (2019). Synchronous and metachronous liver metastases in patients with colorectal cancer-towards a clinically relevant definition. World J Surg Oncol.

[26] Jiang T, Liu S, Wu X, Liu X, Li W, Yang S (2021). Nomogram to predict distant metastasis probability for pathological complete response rectal cancer patients after neoadjuvant chemoradiotherapy. Cancer Manag Res.

[27] Zhou C, Liu HS, Liu XH, Zheng XB, Hu T, Liang ZX (2019). Preoperative assessment of lymph node metastasis in clinically node-negative rectal cancer patients based on a nomogram consisting of five clinical factors. Ann Transl Med.

[28] Liu J, Huang X, Yang W, Li C, Li Z, Zhang C (2020). Nomogram for predicting overall survival in stage II-III colorectal cancer. Cancer Med.

[29] Kim C, Kim WR, Kim KY, Chon HJ, Beom SH, Kim H (2018). Predictive nomogram for recurrence of stage I colorectal cancer after curative resection. Clin Colorectal Cancer.

[30] Borumandnia N, Doosti H, Jalali A, Khodakarim S, Charati JY, Pourhoseingholi MA, Talebi A, Agah S. Nomogram to predict the overall survival of colorectal cancer patients: a multicenter national study. Int J Environ Res Public Health. 2021;18(15):7734. 10.3390/ijerph18157734 PMC8345484 34360026

[31] Meng Q, Zheng N, Wen R, Sui J, Zhang W (2021). Preoperative nomogram to predict survival following colorectal cancer liver metastasis simultaneous resection. J Gastrointest Oncol.

[32] Liu Z, Xu Y, Xu G, Baklaushev VP, Chekhonin VP, Peltzer K (2021). Nomogram for predicting overall survival in colorectal cancer with distant metastasis. BMC Gastroenterol.

[33] Dai S, Ye Y, Kong X, Li J, Ding K (2021). A predictive model for early recurrence of colorectal-cancer liver metastases based on clinical parameters. Gastroenterol Rep (Oxf).

[34] Ding X, Tian S, Hu J, Wang G, Yu X, Fu D (2021). Risk and prognostic nomograms for colorectal neuroendocrine neoplasm with liver metastasis: a population-based study. Int J Color Dis.

[35] Mo S, Cai X, Zhou Z, Li Y, Hu X, Ma X (2020). Nomograms for predicting specific distant metastatic sites and overall survival of colorectal cancer patients: A large population-based real-world study. Clin Transl Med.

[36] Tang M, Wang H, Cao Y, Zeng Z, Shan X, Wang L (2021). Nomogram for predicting occurrence and prognosis of liver metastasis in colorectal cancer: a population-based study. Int J Color Dis.

[37] Sparano JA, Gray RJ, Makower DF, Pritchard KI, Albain KS, Hayes DF (2015). Prospective validation of a 21-gene expression assay in breast cancer. N Engl J Med.

[38] Gorelik E, Landsittel DP, Marrangoni AM, Modugno F, Velikokhatnaya L, Winans MT (2005). Multiplexed immunobead-based cytokine profiling for early detection of ovarian cancer. Cancer Epidemiol Biomark Prev.

[39] Tsai MS, Su YH, Ho MC, Liang JT, Chen TP, Lai HS (2007). Clinicopathological features and prognosis in resectable synchronous and metachronous colorectal liver metastasis. Ann Surg Oncol.

[40] Wang WS, Lin JK, Chiou TJ, Liu JH, Fan FS, Yen CC (2000). Preoperative carcinoembryonic antigen level as an independent prognostic factor in colorectal cancer: Taiwan experience. Jpn J Clin Oncol.

[41] Goldstein M, Mitchell EP (2005). Carcinoembryonic antigen in the staging and follow-up of patients with colorectal cancer. Cancer Investig.

[42] Khan MS, Khan MA, Akbar SA, Bakar MA, Khattak S, Syed AA (2019). Prognostic significance of pre- and post-operative serum carcinoembryonic antigen levels in patients presented with rectal carcinoma; an experience from Shaukat Khanum Memorial Cancer Hospital and Research Center Lahore. J Pak Med Assoc.

[43] Hara M, Kanemitsu Y, Hirai T, Komori K, Kato T (2008). Negative serum carcinoembryonic antigen has insufficient accuracy for excluding recurrence from patients with Dukes C colorectal cancer: analysis with likelihood ratio and posttest probability in a follow-up study. Dis Colon Rectum.

[44] Bockhorn M, Frilling A, Fruhauf NR, Neuhaus J, Molmenti E, Trarbach T (2008). Survival of patients with synchronous and metachronous colorectal liver metastases--is there a difference?. J Gastrointest Surg.

[45] Cho M, Akiba C, Lau C, Smith D, Telatar M, Afkhami M (2016). Impact of RAS and BRAF mutations on carcinoembryonic antigen production and pattern of colorectal metastases. World J Gastrointest Oncol.

[46] Chuang SC, Su YC, Lu CY, Hsu HT, Sun LC, Shih YL (2011). Risk factors for the development of metachronous liver metastasis in colorectal cancer patients after curative resection. World J Surg.

[47] Mohr AM, Gould JJ, Kubik JL, Talmon GA, Casey CA, Thomas P (2017). Enhanced colorectal cancer metastases in the alcohol-injured liver. Clin Exp Metastasis.

[48] Khan MS, Bakar MA, Saba A, Khan MA, Akbar SA, Islam Nasir IU (2019). Risk factors effecting development of metachronous liver metastasis in rectal cancer patients after curative surgical resection. Shaukat Khanum Memorial Cancer Hospital and Research Centre, Lahore experience. J Pak Med Assoc.

[49] Balschun K, Haag J, Wenke AK, von Schonfels W, Schwarz NT, Rocken C (2011). KRAS, NRAS, PIK3CA exon 20, and BRAF genotypes in synchronous and metachronous primary colorectal cancers diagnostic and therapeutic implications. J Mol Diagn.

[50] Tsilimigras DI, Ntanasis-Stathopoulos I, Bagante F, Moris D, Cloyd J, Spartalis E (2018). Clinical significance and prognostic relevance of KRAS, BRAF, PI3K and TP53 genetic mutation analysis for resectable and unresectable colorectal liver metastases: A systematic review of the current evidence. Surg Oncol.

[51] Nakayama I, Hirota T, Shinozaki E. BRAF mutation in colorectal cancers: from prognostic marker to targetable mutation. Cancers (Basel). 2020;12(11):3236. 10.3390/cancers12113236 PMC7694028 33152998

[52] Margonis GA, Buettner S, Andreatos N, Kim Y, Wagner D, Sasaki K (2018). Association of BRAF mutations with survival and recurrence in surgically treated patients with metastatic colorectal liver cancer. JAMA Surg.

[53] Feng Q, Liang L, Ren L, Chen J, Wei Y, Chang W (2015). A specific KRAS codon 13 mutation is an independent predictor for colorectal cancer metachronous distant metastases. Am J Cancer Res.

[54] Mekenkamp LJ, Koopman M, Teerenstra S, van Krieken JH, Mol L, Nagtegaal ID (2010). Clinicopathological features and outcome in advanced colorectal cancer patients with synchronous vs metachronous metastases. Br J Cancer.

[55] Seeberg LT, Brunborg C, Waage A, Hugenschmidt H, Renolen A, Stav I (2017). Survival impact of primary tumor lymph node status and circulating tumor cells in patients with colorectal liver metastases. Ann Surg Oncol.

[56] Filip S, Vymetalkova V, Petera J, Vodickova L, Kubecek O, John S, Cecka F, Krupova M, Manethova M, Cervena K, Vodicka P. Distant metastasis in colorectal cancer patients-do we have new predicting clinicopathological and molecular biomarkers? A comprehensive review. Int J Mol Sci. 2020;21(15):5255. 10.3390/ijms21155255 PMC7432613 32722130

[57] Li Destri G, La Greca G, Pesce A, Conti E, Puleo S, Portale TR (2019). Lymph node ratio and liver metachronous metastases in colorectal cancer. Ann Ital Chir.

[58] de Ridder JA, Knijn N, Wiering B, de Wilt JH, Nagtegaal ID (2015). Lymphatic invasion is an independent adverse prognostic factor in patients with colorectal liver metastasis. Ann Surg Oncol.

[59] Dekker E, Tanis PJ, Vleugels JLA, Kasi PM, Wallace MB (2019). Colorectal cancer. Lancet.

[60] Nerad E, Lahaye MJ, Maas M, Nelemans P, Bakers FC, Beets GL (2016). Diagnostic accuracy of CT for local staging of colon cancer: a systematic review and meta-analysis. AJR Am J Roentgenol.

